# Supplementary material for: A multimodal cross-species comparison of pancreas development
Source: Nat Commun. 2025 Oct 22;16:9355. doi: 10.1038/s41467-025-64774-4 (PMC12546597; doi:10.1038/s41467-025-64774-4)
Supplement: Supplementary file 1 — Supplementary Information [file 41467_2025_64774_MOESM1_ESM.pdf]

Supplementary information

A multimodal cross-species comparison of pancreas development

Kaiyuan Yang[1,2,†], Hannah Spitzer[3,4,†], Michael Sterr[1,2], Karin Hrovatin[3,5], Sean de la O[6,7,8], Xinghao Zhang[9,10], Eunike Sawitning Ayu Setyono[1,2], Minhaz Ud-Dean[11], Thomas Walzthoeni[11], Krzysztof Flisikowski[12], Tatiana Flisikowska[12], Angelika Schnieke[12], Katharina Scheibner[1,2], James M. Wells[9,10,13], Julie B. Sneddon[6,7,8], Barbara Kessler[2,14,15], Eckhard Wolf[2,14,15], Elisabeth Kemter[2,14,15], Fabian J. Theis[3,5,16*], Heiko Lickert[1,2,17*]

[1] Institute of Diabetes and Regeneration Research (IDR), Helmholtz Munich, Neuherberg, Germany.
[2] German Center for Diabetes Research (DZD), Neuherberg, Germany.
[3] Institute of Computational Biology (ICB), Helmholtz Munich, Neuherberg, Germany.
[4] Institute for Stroke and Dementia Research, University Hospital, Ludwig Maximilian University of Munich, Munich, Germany.
[5] Department of Mathematics, Technical University of Munich, Munich, Germany.
[6] Department of Cell and Tissue Biology, University of California, San Francisco, USA.
[7] Diabetes Center, University of California, San Francisco, USA.
[8] Eli and Edythe Broad Center of Regeneration Medicine and Stem Cell Research, University of California, San Francisco, USA.
[9] Division of Developmental Biology, Cincinnati Children's Hospital Medical Center, Cincinnati, USA.
[10] Center for Stem Cell and Organoid Medicine (CuSTOM), Cincinnati Children's Hospital Medical Center, Cincinnati, USA
[11] Core Facility Genomics, Helmholtz Munich, Neuherberg, Germany.
[12] Chair of Livestock Biotechnology, Department of Molecular Life Sciences, School of Life Sciences, Technical University of Munich, Freising, Germany.
[13] Division of Endocrinology, Cincinnati Children's Hospital Medical Center, Cincinnati, USA.
[14] Chair for Molecular Animal Breeding and Biotechnology Gene Center, Ludwig Maximilian University of Munich, Munich, Germany.
[15] Center for Innovative Medical Models (CiMM), Ludwig Maximilian University of Munich, Munich, Germany.
[16] School of Life Sciences Weihenstephan, Technical University of Munich, Freising, Germany.
[17] School of Medicine, Technical University of Munich, Munich, Germany.
† These authors contributed equally: Kaiyuan Yang, Hannah Spitzer.
*Correspondence to
heiko.lickert@helmholtz-munich.de and fabian.theis@helmholtz-munich.de

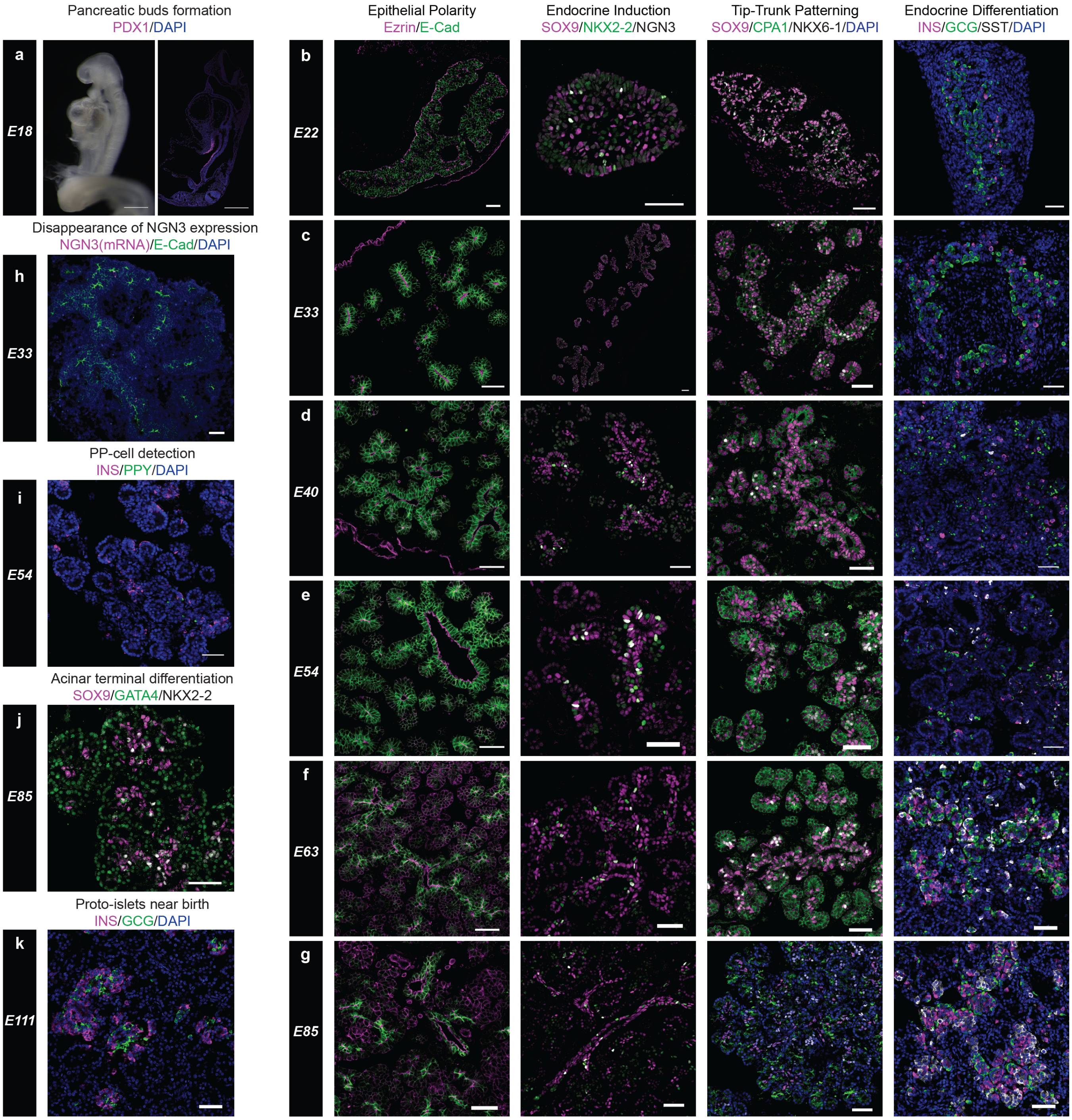

Supplementary Fig. 1: Characterization of pig pancreas organogenesis.

a. Immunofluorescence identification of pancreatic buds emerging at E18 (bright field image, scale bar 1 mm; immunostaining, scale bar 500 µm). **b-g.** During E22-85, markers of epithelial polarity, endocrine induction, tip-trunk patterning, and endocrine differentiation are shown in pancreas sections at the corresponding ages. **h.** NEUROG3 mRNA is detected weakly expressed in 4 cells in E33 pancreas by RNAscope. **i.** PPY$^+$ PP cells appear in E54 pancreas. **j.** Acinar, ductal and endocrine TFs show exclusive expression at E85. **k.** Endocrine cells form

proto-islets 3-day before birth (E111). Scale bar 50 µm. Wild-type pig samples are used for this figure. Images are representative of 3 pig pancreas samples per time point.

a

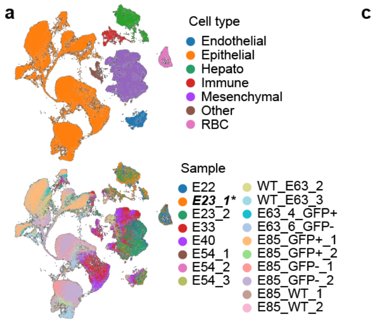

** Sample E23_1 was excluded from analysis due to unsuccessful dissection, as scRNA-seq confirmed it contained only 6 ductal cells*

c

Sample	Number of pancreas	Acinar	Alpha	Beta	Delta	Ductal	Endothelial	FEV	Hepato	Immune	MPC	Mesenchymal	NGN3	Other	PP	PEC	RBC
E22	6	1	105	34	4	31	522	1	1730	322	267	6110	20	48	0	5	2215
E23_1*	4	0	0	0	0	6	533	0	3982	694	0	2476	0	10	0	0	278
E23_2	4	0	52	43	1	8	517	3	1022	291	155	5300	10	32	0	70	260
E33	4	3	657	324	7	1191	648	16	0	147	3716	6185	2	990	1	34	75
E40	2	4	213	141	4	1503	695	5	0	71	1847	2195	32	423	0	12	0
E54_1	1	214	118	145	15	344	13	13	1	6	11	55	28	16	6	16	0
E54_2	1	255	113	195	28	210	27	12	0	8	9	52	31	11	6	19	1
E54_3	1	272	96	182	29	284	15	9	0	18	35	73	39	12	9	16	0
E63_4_GFP+	2	295	768	2078	224	121	0	14	0	43	3	13	56	2	335	57	0
E63_6_GFP-		2157	7	14	9	1010	1	4	0	2	3	40	141	1	5	4	0
WT_E63_2	1	1603	223	379	89	462	117	12	0	153	7	908	86	81	30	13	3
WT_E63_3	1	2486	95	311	57	918	0	15	0	3	15	15	206	1	34	27	0
E85_GFP+_1	1	94	1722	5308	785	804	1	290	0	94	4	3	49	1	545	39	0
E85_GFP-_1		3802	44	34	31	4689	4	170	0	11	7	3	188	0	63	2	0
E85_GFP+_2	1	76	2136	5603	864	188	3	727	0	24	6	0	53	0	1074	51	0
E85_GFP-_2		6040	45	33	47	3060	3	790	0	13	60	6	352	0	261	4	0
E85_WT_1	1	2786	261	586	113	542	164	122	0	140	1	236	68	30	65	9	2
E85_WT_2	1	2559	1078	2609	511	2492	5	842	0	22	1	3	251	0	426	18	0

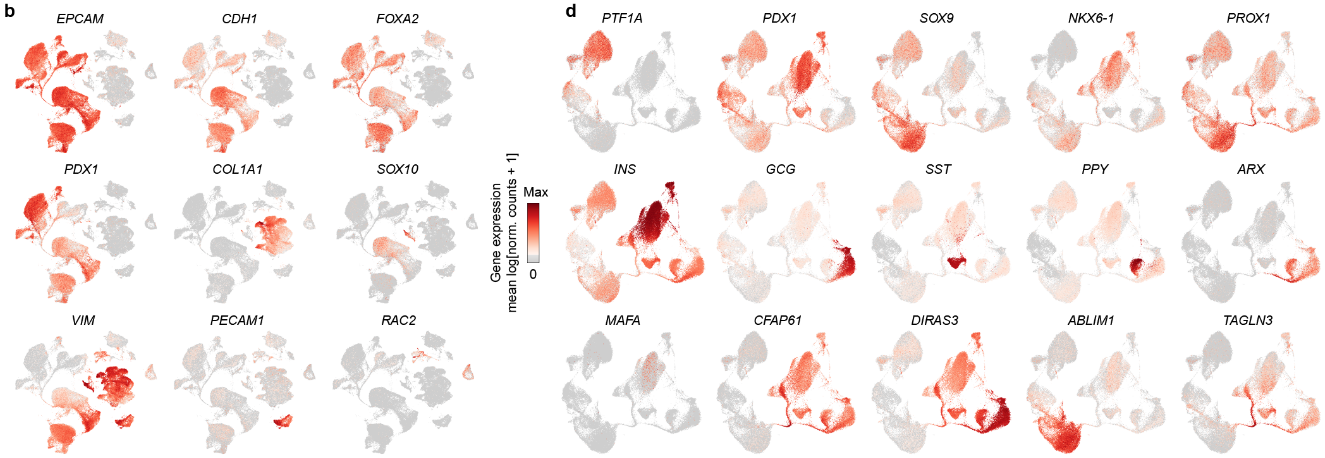

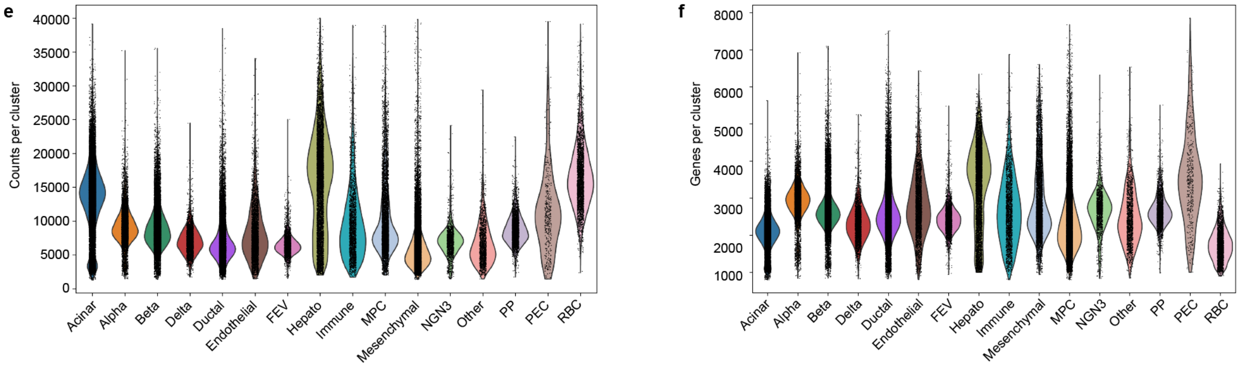

Supplementary Fig. 2: Composition of the integrated pig scRNA-seq pancreas atlas.
a. UMAPs showing non-epithelial and epithelial cell clusters (top) and cell type counts per samples (bottom) of the integrated pig scRNA-seq dataset. Hepato, hepatocytes; RBC, red blood cells. **b.** UMAPs showing known marker gene expression of the non-epithelial and epithelial cell clusters. **c.** Table summarizing pancreas number and cell counts in identified cell types of each sample shown in **a** (bottom). **d**. UMAPs showing known marker gene expression of cell clusters identified in the pig pancreas atlas. **e,f.** Violin plots showing distribution of the detected read counts (**e**) and gene counts (**f**) across clusters (Supplementary Data 13). Data from scRNA-seq of pancreatic cells from wild-type and *INS*-eGFP pigs. Detailed sample information is provided in Supplementary Data 1.

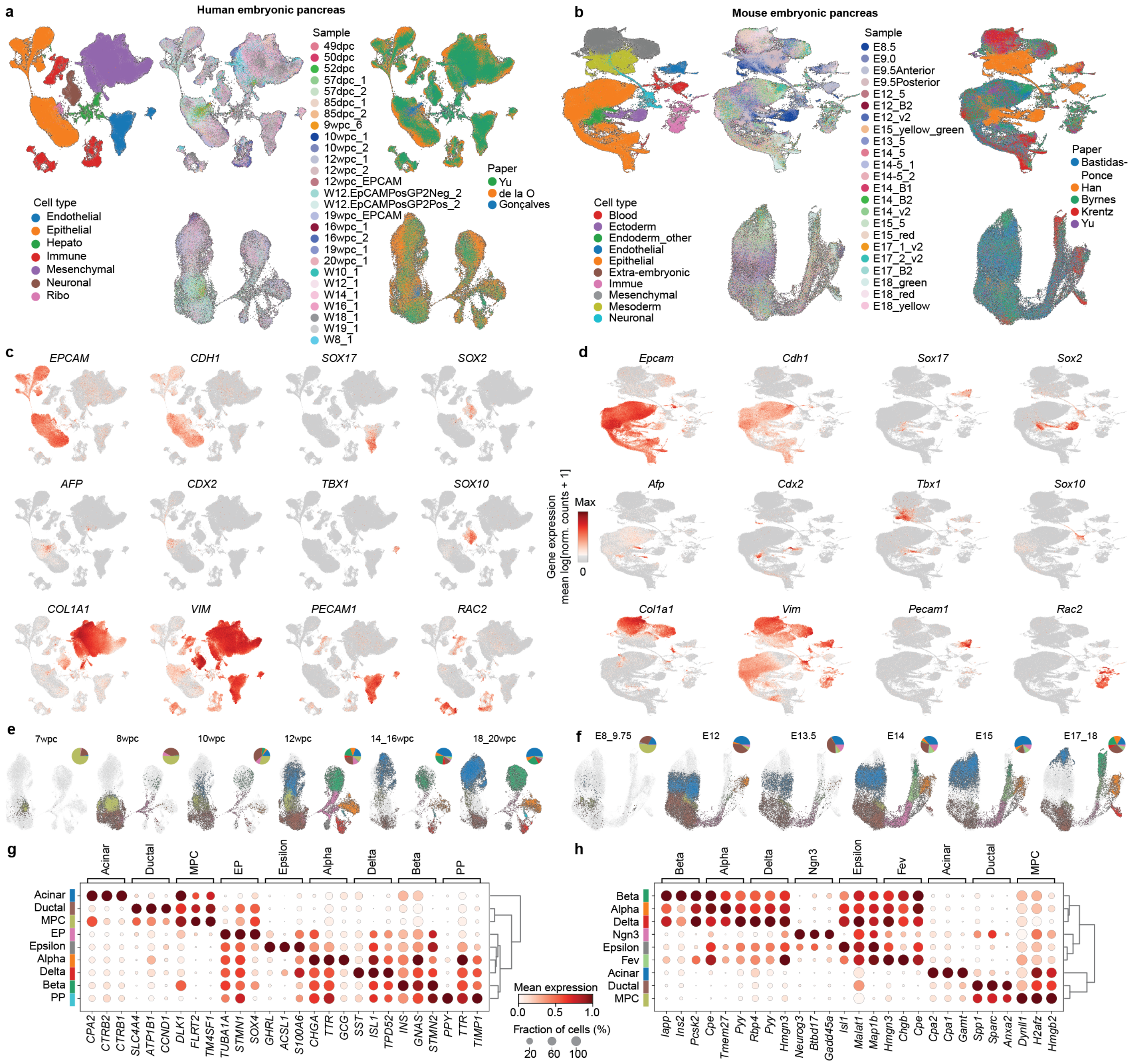

Supplementary Fig. 3: Composition of the human and mouse integrated scRNA-seq pancreas atlas.

a,b. UMAPs showing all cell type (top panel) and epithelial cell (bottom panel) clusters, sample distribution, and data sources of the integrated scRNA-seq datasets of human (**a**) and mouse (**b**) pancreas development. Hepato, hepatocytes; Ribo, ribosomal gene-enriched cells. **c,d**. UMAPs showing known marker gene expression of the non-epithelial and epithelial cell clusters in human (**c**) and mouse (**d**). **e,f**. UMAPs showing cluster changes at different developmental stages in human (**e**) and mouse (**f**). Cell clusters are highlighted and colored by cell type, with a pie chart of relative cell type composition at the upper right corner. **g,h**. Dot plots showing mean gene expression of marker genes for each cluster of the integrated human (**g**) and mouse (**h**) pancreas atlases (Supplementary Data 2).

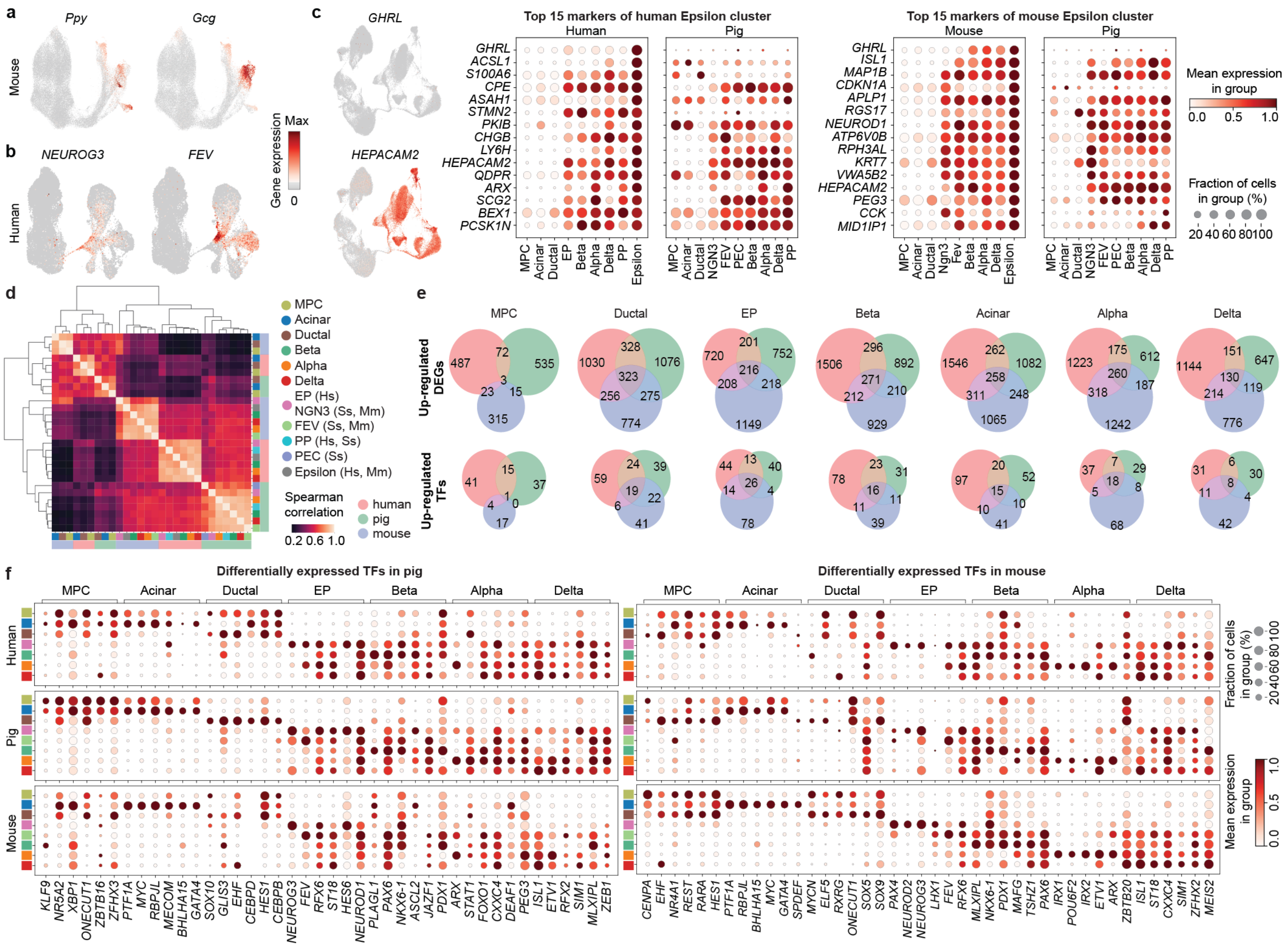

Supplementary Fig. 4: Comparison of cell-type specific transcriptional profiles using human, pig, and mouse pancreas atlases.

a. UMAPs of *Ppy* and *Gcg* expression in mouse pancreas atlas. **b**. UMAPs of *NEUROG3* and *FEV* expression in human pancreas atlas. **c**. (Left) UMAPs of *GRHL* and *HEPACAM2* expression in pig pancreas atlas. (Middle) dot plots showing the top 15 marker genes of the human Epsilon clusters visualized in all clusters for human and pig. (Right) dot plots showing the top 15 marker genes of the mouse Epsilon clusters visualized in all clusters for mouse and pig. **d**. Spearman correlation of mean counts per cluster for each gene between human, pig and mouse. Gene counts from pig and mouse were mapped to human using orthologues. Genes were reduced to the intersection (n=851) of 4000 highly variable genes for all species. **e**. Venn diagrams showing overlapping and distinct up-regulated DEGs (FDR-adjusted p-value < 0.05, log-fold change >0.75, Supplementary Data 3) of each cluster for all three species (top panel, all up-regulated DEGs; bottom panel, up-regulated TFs), labeled with gene numbers and colored by species as shown in d. **f**. Dot plots showing mean expression of differentially expressed TFs in pig (left) and mouse (right), visualized in all clusters (square color = cell type color as shown in d) for all three species (one-vs-rest, computed with edgeR, Supplementary Data 3). Shown were genes with the highest log-fold change and expressed in more than 20% of cells of the cluster. Gene names were mapped to human genes using orthologues. Dot size shows fraction of cells in cluster on a logarithmic scale. Data from scRNA-seq of pancreatic cells from wild-type and *INS*-eGFP pigs. Detailed sample information is provided in Supplementary Data 1.

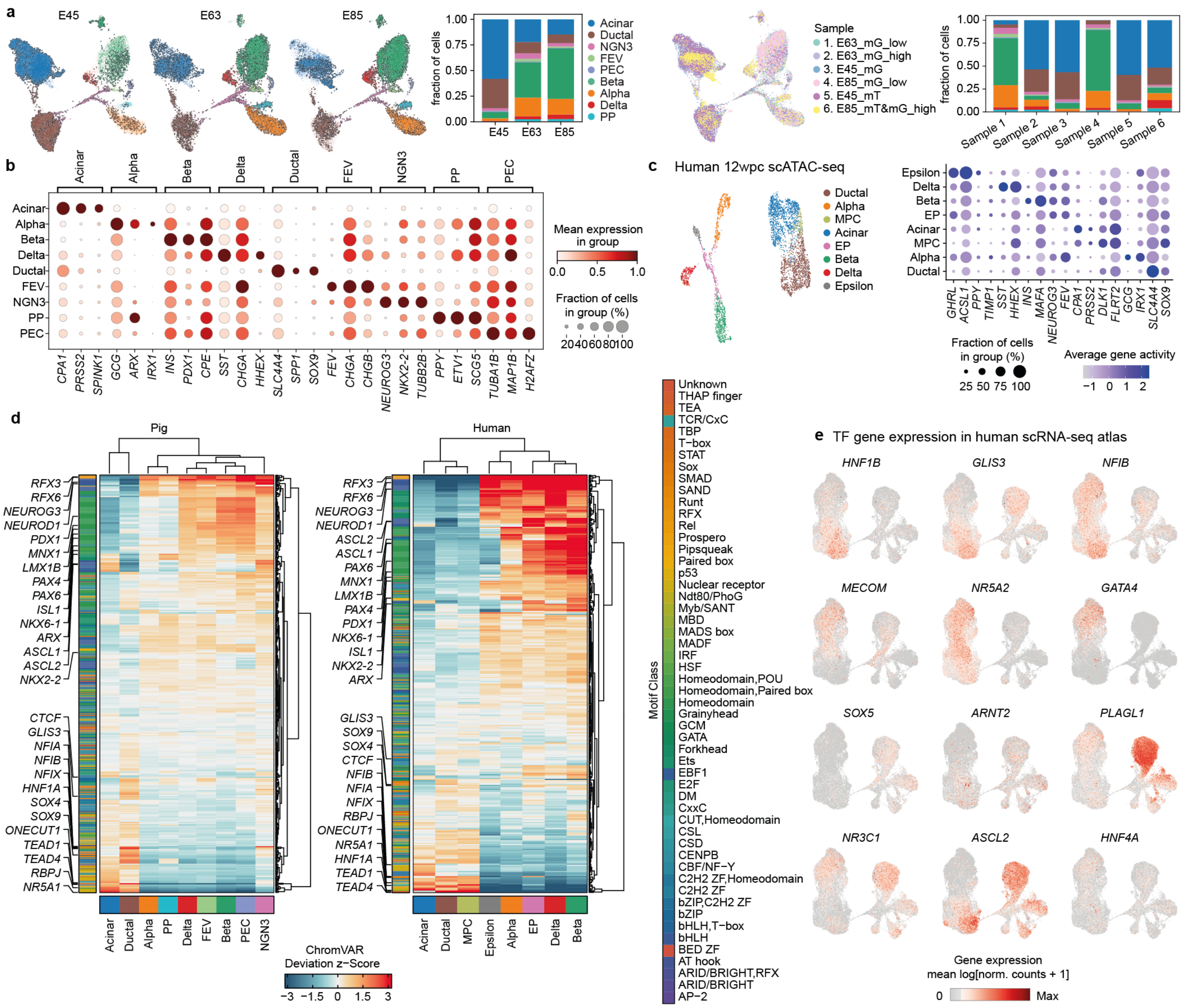

Supplementary Fig. 5: Comparison of joint transcriptional and chromatin accessibility profiles in pig and human pancreas atlas.

a. (Left panel) UMAPs showing cluster changes at different developmental stages of the pig multiome pancreas atlas with a bar plot summarizing cell cluster distribution per age. (Right panel) UMAP showing sample distribution of the pig multiome pancreas atlas with a bar plot summarizing cell cluster distribution per sample. **b**. Dot plots showing mean gene expression of marker genes for each cluster of the pig multiome pancreas atlas. **c**. UMAP of cell clusters in human 12wpc scATAC-seq dataset[28] with a dot plot showing inferred top gene activities for each cluster. **d**. Heatmap of all differentially active motifs across cell types computed with chromVAR using pig multiome and human 12wpc scATAC-seq datasets. **e**. UMAPs showing the expression of representative active TF motifs in the integrated human scRNA-seq atlas. Data from Multiome analysis of pancreatic cells from PTF1A-codon-improved-Cre/ROSA-mTmG pigs. Detailed sample information is provided in Supplementary Data 1.

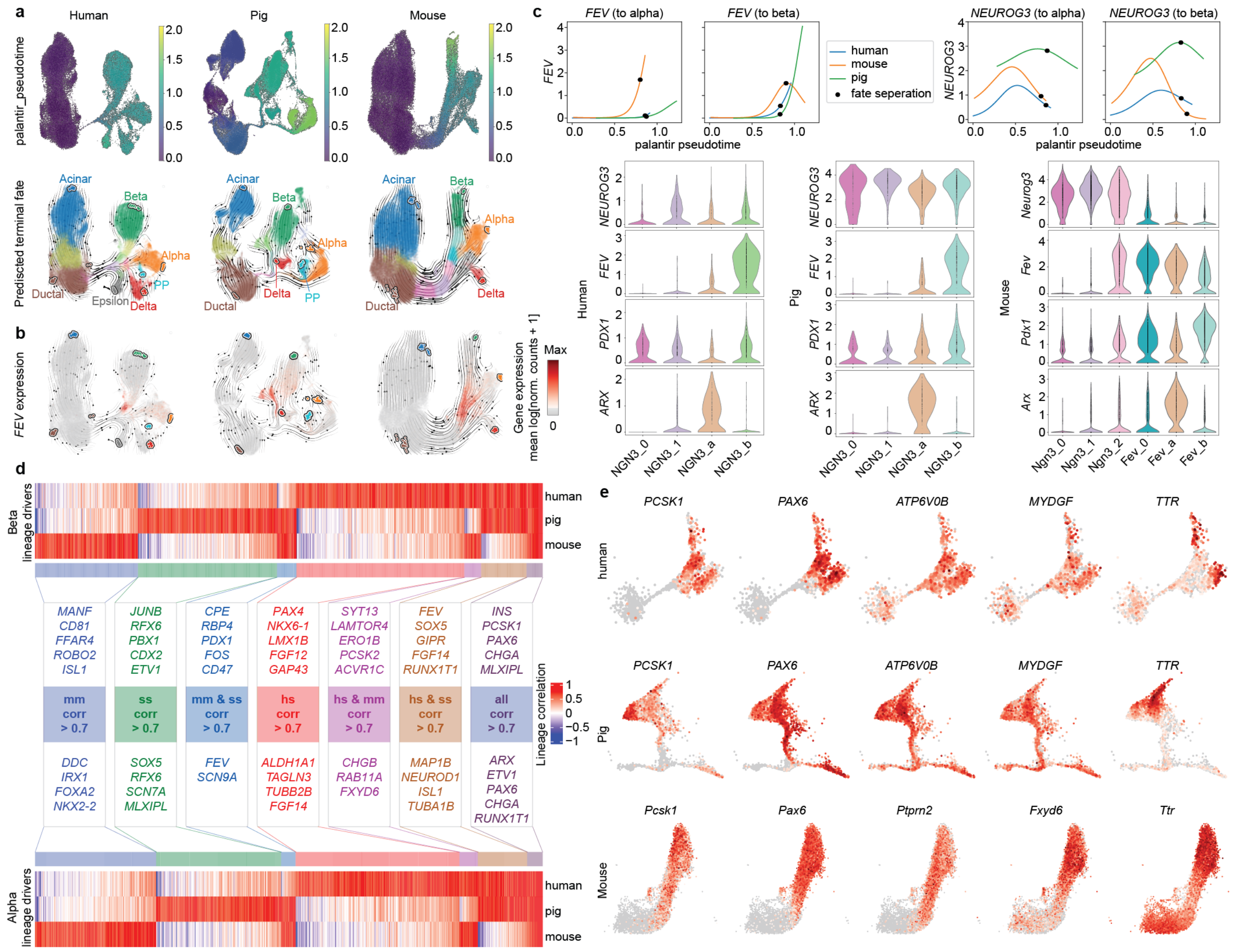

Supplementary Fig. 6: Comparison of endocrinogenesis in human, pig and mouse.

a. UMAPs showing Palantir-pseudotime (top panel) and CellRank-predicted terminal states (bottom panel) in human, pig and mouse. **b**. UMAPs of *FEV* expression in all three species overlaid with CellRank-inferred terminal states and trajectories (arrows). **c**. (Top panel) line plots of *FEV* and *NEUROG3* expression along Palantir-pseudotime in all three species with black dots indicating the time of alpha and beta fate separation. (Bottom panel) violin plots showing *NEUROG3, FEV, PDX1* and *ARX* expression of the endocrine progenitor subclusters towards alpha and beta lineages. **d**. Heatmap showing lineage correlation scores of the union of positive lineage drivers from each species. Shown gene examples had a correlation score > 0.7 and were colored according to species overlapping indicated in the middle panel. (mm, mouse; ss, pig; hs, human; corr, correlation) **e**. UMAPs of beta and alpha lineage driver gene expression in human, pig and mouse. Data from scRNA-seq of pancreatic cells from wild-type and *INS*-eGFP pigs. Detailed sample information is provided in Supplementary Data 1.

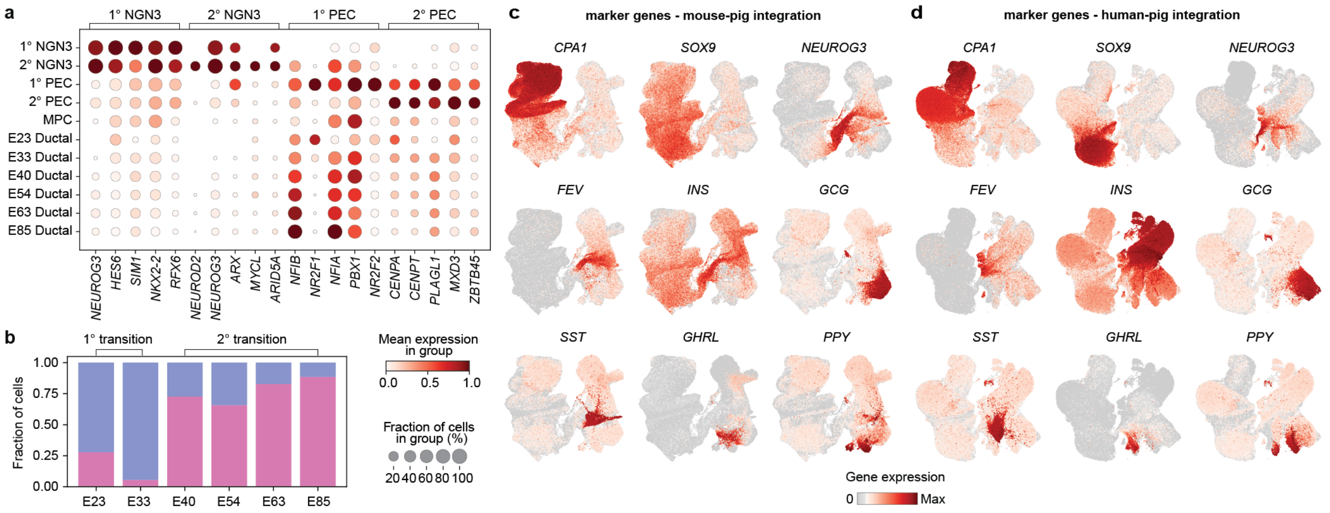

Supplementary Fig. 7: PEC exists as a potential endocrine progenitor population.

a. Dot plot of the top 5 differentially expressed TFs from Fig. 5d visualized additionally in MPC and stage-wise Ductal clusters. **b**. Bar plot showing distribution of NGN3 and PEC clusters at the corresponding developmental stage. **c**, **d**. UMAPs showing established pancreatic cell type markers in (**c**) sysVI-integrated mouse-pig scRNA-seq pancreas atlases and (**d**) sysVI-integrated human-pig scRNA-seq pancreas atlases. Data from scRNA-seq of pancreatic cells from wild-type and *INS*-eGFP pigs. Detailed sample information is provided in Supplementary Data 1.

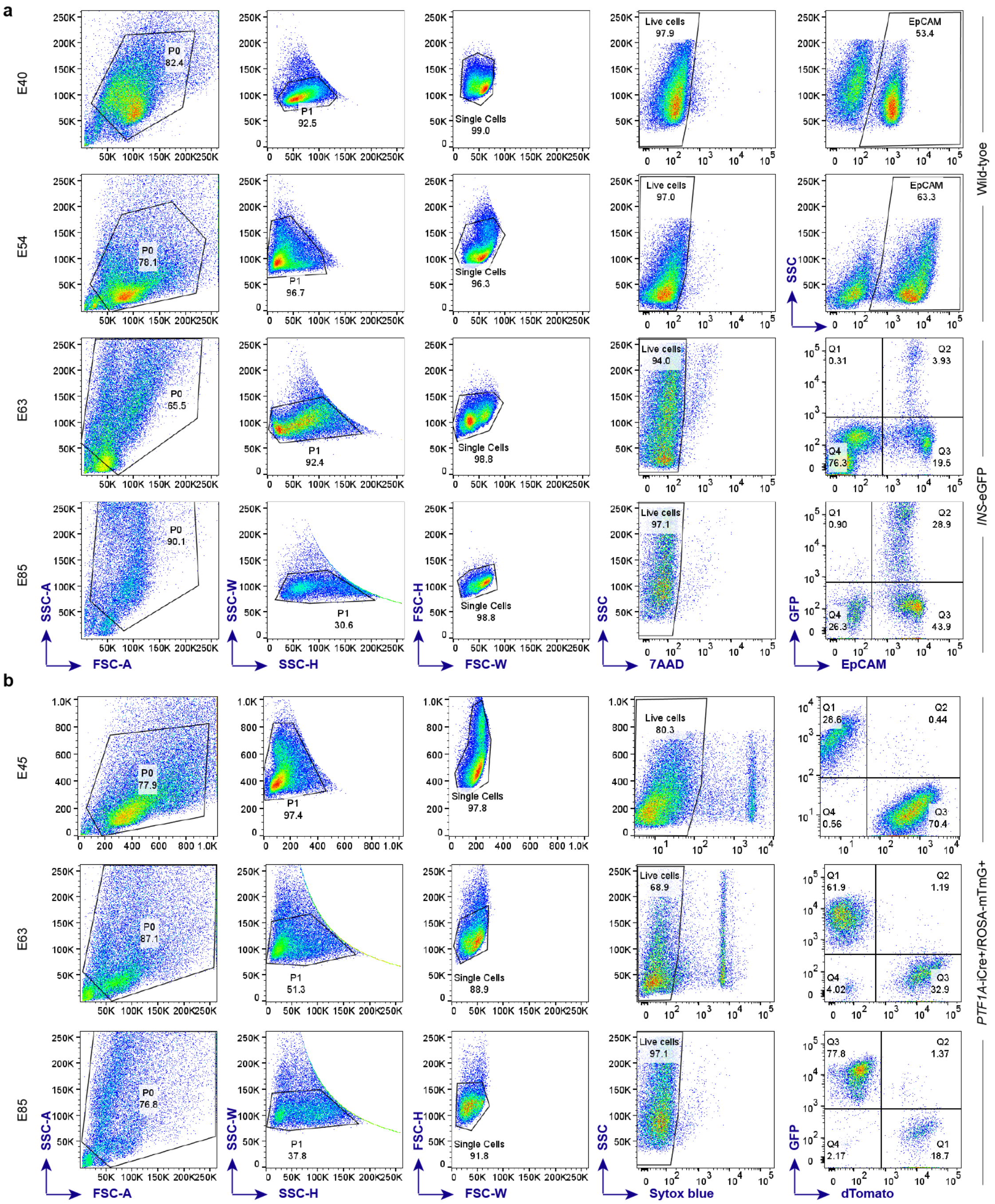

Supplementary Fig. 8: Gating strategy for pancreatic epithelial cell enrichment.

a. Flow cytometry plots for the enrichment of live pancreatic epithelial cell from wild-type and *INS*-eGFP pig embryos. Cells were sequentially gated for single cells (based on FSC-A/SSC-A, SSC-H/SSC-W, and FSC-H/FSC-W), live cells (7AAD-negative), and epithelial cells (EpCAM-positive). Beta cells were subsequently enriched based on *INS*-eGFP fluorescence. **b**. Flow cytometry plots for the enrichment of live pancreatic epithelial cells from *PTF1A*-iCre^{+}/ROSA-mTmG^{+} embryos. Following single-cell and live-cell (Sytox Blue-negative) gating, pancreatic epithelial cells were isolated based on constitutive Cre recombinase-induced GFP expression. FSC, forward scatter; SSC, side scatter. Plots are representative of all available pig pancreas samples per time point as detailed in Supplementary Data 1.

Description of Additional Supplementary Files

Supplementary Data 1: Overview of the next-generation sequencing samples in this study.

Supplementary Data 2: Marker genes of cell clusters in the human, pig and mouse pancreas integrated scRNA-seq datasets.

Supplementary Data 3: Differentially expressed genes of each cell cluster in the human, pig and mouse pancreas integrated scRNA-seq datasets.

Supplementary Data 4: CellRank computed lineage drivers and enriched pathways for beta and alpha lineage in human, pig and mouse.

Supplementary Data 5: Comparison of NEUROG3 targets derived from pig/human pancreas multiome atlases and human stem cell models.

Supplementary Data 6: Differentially expressed genes and enriched pathways of the pig and human beta subclusters.

Supplementary Data 7: CellOracle inferred GRNs of the pig beta subclusters.

Supplementary Data 8: Differentially expressed genes and enriched pathways comparing between lineages by tradeSeq.

Supplementary Data 9: Differentially expressed genes of the pig endocrine progenitor subpopulations during 1° and 2° transition.

Supplementary Data 10: Antibodies and hiPSC differentiation protocol used in this study.

Supplementary Data 11: Improved genome annotation for *Sus scrofa* showing UMI counts for 37 genes of interest before and after gene extension.

Supplementary Data 12: Thresholds used for low quality cell filtering in the preprocessing of scRNA-seq, scATAC-seq and multiome raw data.

Supplementary Data 13: Read counts and gene counts across clusters of pig scRNA-seq data.

Supplementary Data 14: Differentially expressed genes of pig PEC cluster compared to other clusters.
