# Supplementary material for: A multimodal cross-species comparison of pancreas development
Source: Nat Commun. 2025 Oct 22;16:9355. doi: 10.1038/s41467-025-64774-4 (PMC12546597; doi:10.1038/s41467-025-64774-4)
Supplement: Supplementary file 3 — Description of Additional Supplementary Files [file 41467_2025_64774_MOESM3_ESM.pdf]

Supplementary Data 1: Overview of the next-generation sequencing samples in this study.

Supplementary Data 2: Marker genes of cell clusters in the human, pig and mouse pancreas integrated scRNA-seq datasets.

Supplementary Data 3: Differentially expressed genes of each cell cluster in the human, pig and mouse pancreas integrated scRNA-seq datasets.

Supplementary Data 4: CellRank computed lineage drivers and enriched pathways for beta and alpha lineage in human, pig and mouse.

Supplementary Data 5: Comparison of NEUROG3 targets derived from pig/human pancreas multiome atlases and human stem cell models.

Supplementary Data 6: Differentially expressed genes and enriched pathways of the pig and human beta subclusters.

Supplementary Data 7: CellOracle inferred GRNs of the pig beta subclusters.

Supplementary Data 8: Differentially expressed genes and enriched pathways comparing between lineages by tradeSeq.

Supplementary Data 9: Differentially expressed genes of the pig endocrine progenitor subpopulations during 1° and 2° transition.

Supplementary Data 10: Antibodies and hiPSC differentiation protocol used in this study.

Supplementary Data 11: Improved genome annotation for *Sus scrofa* showing UMI counts for 37 genes of interest before and after gene extension.

Supplementary Data 12: Thresholds used for low quality cell filtering in the preprocessing of scRNA-seq, scATAC-seq and multiome raw data.

Supplementary Data 13: Read counts and gene counts across clusters of pig scRNA-seq data.

Supplementary Data 14: Differentially expressed genes of pig PEC cluster compared to other clusters.
