# Supplementary material for: A multimodal cross-species comparison of pancreas development
Source: Nat Commun. 2025 Oct 22;16:9355. doi: 10.1038/s41467-025-64774-4 (PMC12546597; doi:10.1038/s41467-025-64774-4)
Supplement: Supplementary file 18 — Reporting Summary [file 41467_2025_64774_MOESM18_ESM.pdf]

Corresponding author(s): Heiko Lickert & Fabian Theis

Last updated by author(s): Sep 10, 2025

Reporting Summary

Nature Portfolio wishes to improve the reproducibility of the work that we publish. This form provides structure for consistency and transparency in reporting. For further information on Nature Portfolio policies, see our Editorial Policies and the Editorial Policy Checklist.

Statistics

For all statistical analyses, confirm that the following items are present in the figure legend, table legend, main text, or Methods section.

n/a	Confirmed	
☐	☒	The exact sample size (*n*) for each experimental group/condition, given as a discrete number and unit of measurement
☐	☒	A statement on whether measurements were taken from distinct samples or whether the same sample was measured repeatedly
☐	☒	The statistical test(s) used AND whether they are one- or two-sided<br>*Only common tests should be described solely by name; describe more complex techniques in the Methods section.*
☐	☒	A description of all covariates tested
☐	☒	A description of any assumptions or corrections, such as tests of normality and adjustment for multiple comparisons
☐	☒	A full description of the statistical parameters including central tendency (e.g. means) or other basic estimates (e.g. regression coefficient) AND variation (e.g. standard deviation) or associated estimates of uncertainty (e.g. confidence intervals)
☐	☒	For null hypothesis testing, the test statistic (e.g. *F*, *t*, *r*) with confidence intervals, effect sizes, degrees of freedom and *P* value noted<br>*Give P values as exact values whenever suitable.*
☐	☒	For Bayesian analysis, information on the choice of priors and Markov chain Monte Carlo settings
☐	☒	For hierarchical and complex designs, identification of the appropriate level for tests and full reporting of outcomes
☐	☒	Estimates of effect sizes (e.g. Cohen's *d*, Pearson's *r*), indicating how they were calculated

Our web collection on statistics for biologists contains articles on many of the points above.

Software and code

Policy information about availability of computer code

Data collection	Zeiss Zen 2.3 lite (Blue edition)
Data analysis	All softwares for data analysis were described in methods section of the manuscript. Software packages used are as follows: gffcompare (v0.12.1), CellRanger (v3.1.1), CellRangerARC pipeline (v 2.0.0), Scanpy (v1.8.2 and 1.9.1), Scran (v1.22.1), DropletUtils (v1.14.2), Scanorama(v1.7.1), scvi-tools (v1.3.3), scrublet (v0.2.3), DoubletDetection (v4.2), scds (v1.10.0), scDblFinder (v1.11.4), DoubletFinder (v2.0.3), Signac (v1.9.0), scANVI (v0.20.3), Muon (v0.1.2), SOLO (in scvi-tools v0.19.0), GenomicRanges (v1.46.1), SCTransform (v0.3.3), scry (v1.6.0), scikit learn (v0.24.2), harmonypy (v0.0.9), PoissonVI (in scvi-tools v 0.20.0), edgeR (v4.0.16), CellRank (v1.5.1), Palantir (v1.0.1), tradeSeq (v1.14.0), CellOracle (v0.12.0), SEACells (v0.3.2), Pando (v1.0.3), chromVAR (v1.24.0), MOSCOT (v0.3.3), scGLUE (v0.4.0), Bedtools (v2.29.2), CSBB (v3.0), MACS2 (v2.1.0), GREAT (v4.0.4) ,HOMER (v4.11), ChIPseeker (v1.38.0), ChIPpeakAnno111 (v3.36.1), DESeq2 (v1.42.1).<br>Jupyter notebooks to reproduce the analysis and figures will be made available at https://github.com/theislab/pig-embryo-ana

For manuscripts utilizing custom algorithms or software that are central to the research but not yet described in published literature, software must be made available to editors and reviewers. We strongly encourage code deposition in a community repository (e.g. GitHub). See the Nature Portfolio guidelines for submitting code & software for further information.

Data

Policy information about availability of data

All manuscripts must include a data availability statement. This statement should provide the following information, where applicable:

- Accession codes, unique identifiers, or web links for publicly available datasets
- A description of any restrictions on data availability
- For clinical datasets or third party data, please ensure that the statement adheres to our policy

The data generated in this study have been deposited in NCBI's Gene Expression Omnibus. The pig pancreas data is accessible through GEO Series accession number GSE262280 (https://www.ncbi.nlm.nih.gov/geo/query/acc.cgi?acc=GSE262280). The hESC datasets are accessible through GSE261950 (https://www.ncbi.nlm.nih.gov/geo/query/acc.cgi?acc=GSE261950), GSE261951 (https://www.ncbi.nlm.nih.gov/geo/query/acc.cgi?acc=GSE261951), and GSE261952 (https://www.ncbi.nlm.nih.gov/geo/query/acc.cgi?acc=GSE261952). Sequencing data from pig pancreas were aligned using the Sscrofa11.1 assembly of the pig genome (https://www.ebi.ac.uk/ena/browser/view/GCA_000003025.6) and the improved annotation based on the Ensembl annotation version 101 (see Methods; Improved pig gene annotation file will be made available at https://github.com/theislab/pig-embryo-ana). Sequencing data from hESC were aligned using the GRCh37/hg19 reference genome and Ensembl gene annotation (https://www.ncbi.nlm.nih.gov/datasets/genome/GCF_000001405.13/). Source data will be provided. Any other data supporting the findings of this study are available from the corresponding authors on reasonable request.

Research involving human participants, their data, or biological material

Policy information about studies with human participants or human data. See also policy information about sex, gender (identity/presentation), and sexual orientation and race, ethnicity and racism.

Reporting on sex and gender	No findings related to sex or gender were made or presented in this study
Reporting on race, ethnicity, or other socially relevant groupings	No findings related to race, ethnicity, or other groupings were made or presented in this study
Population characteristics	No human participants were involved in this study
Recruitment	No participants were recruited for this study
Ethics oversight	The Ethics Committee of the Technical University Munich

Note that full information on the approval of the study protocol must also be provided in the manuscript.

Field-specific reporting

Please select the one below that is the best fit for your research. If you are not sure, read the appropriate sections before making your selection.

☒ Life sciences ☐ Behavioural & social sciences ☐ Ecological, evolutionary & environmental sciences

For a reference copy of the document with all sections, see nature.com/documents/nr-reporting-summary-flat.pdf

Life sciences study design

All studies must disclose on these points even when the disclosure is negative.

Sample size	Sample sizes were not predetermined by statistical methods but were chosen based on community standards for exploratory genomics. A sample size of n=3 biological replicates is widely considered sufficient for exploratory sequencing studies like ours, as the depth of data (thousands of cells per sample) allows for robust identification of cell types and states. Practically, for embryonic pig pancreas collection, we aimed to use a minimum of 3 embryos from a single pregnancy whenever possible. This is to minimize animal sacrifice, in full compliance with the German Animal Welfare Act and EU Directive 2010/63/EU.
Data exclusions	During sequencing data processing, low quality cells and cells with unclear identities were excluded to maintain high quality datasets for downstream analysis to derive meaningful results.
Replication	Our standard for biological replication is a minimum of n=3. The specific exceptions are due to the limited availability of embryos: 1) E40 scRNA-seq and E45 multiome samples have n=2 each. Their transcriptional profiles align robustly. This strong correlation across two independent experimental assays performed on similar developmental stages provides high confidence in the results; 2) The E85 multiome sample has n=1. Its profile is faithfully recapitulated in a larger set (n=4) of scRNA-seq replicates from the same stage. Based on this high degree of concordance across assays and stages, we confirm that all major biological findings in the study are reproducible.
Randomization	Randomization is not applicable to this study design as the sample groups are defined by inherent biological characteristics, i.e. embryonic age and species. Our study aims to characterize and compare the normal developmental process rather than to test a hypothesis between randomized groups.
Blinding	Blinding is not relevant to our study because the analytical process is unsupervised and data-driven. The algorithms used to identify cell types and states (like clustering and trajectory inference) are based on the intrinsic patterns within the data itself rather than on pre-defined labels of "sample group".

Reporting for specific materials, systems and methods

We require information from authors about some types of materials, experimental systems and methods used in many studies. Here, indicate whether each material, system or method listed is relevant to your study. If you are not sure if a list item applies to your research, read the appropriate section before selecting a response.

Materials & experimental systems

n/a	Involved in the study
☐	☒ Antibodies
☐	☒ Eukaryotic cell lines
☒	☐ Palaeontology and archaeology
☐	☒ Animals and other organisms
☒	☐ Clinical data
☒	☐ Dual use research of concern
☒	☐ Plants

Methods

n/a	Involved in the study
☐	☒ ChIP-seq
☒	☐ Flow cytometry
☒	☐ MRI-based neuroimaging

Antibodies

Antibodies used

Primary antibodies
Target, Species, Company, Catalogue #, Clone #, RRID, Dilution
Insulin, Mouse, Sigma, I2018, K36AC10, AB_260137, 1:800
Insulin, Rabbit, Cell signaling, 3014, C27C9, AB_2126503, 1:400
Insulin, Guinea pig, Bio-Rad, 5330-0104G, polyclonal, AB_1605150, 1:800
Glucagon, Mouse, Sigma, G2654, K79bB10, AB_259852, 1:500
Glucagon, Rabbit, Cell Signaling, 8233, D16G10, AB_10859908, 1:400
Somatostatin, Mouse, Santa Cruz, sc-55565, G-10, AB_831726, 1:300
Somatostatin, Mouse, Santa Cruz, sc-74556, H-11, AB_2271061, 1:300
Pancreatic Polypeptide, Goat, Sigma, SAB2500747, polyclonal, AB_10611538, 1:300
Ghrelin, Goat, Santa Cruz, sc-10368, polyclonal, AB_2232479, 1:500
Ghrelin, Goat, Santa Cruz, sc-293422, polyclonal, NA, 1:500
GATA4, Goat ,Santa Cruz, sc-1237, polyclonal, AB_2108747, 1:100
PDX1, Goat, R&D Systems, AF2419, polyclonal, AB_355257, 1:500
PDX1, Rabbit, Cell Signaling, 5679, D59H3, AB_10706174, 1:500
SOX9, Rabbit, Millipore, AB5535, polyclonal, AB_2239761, 1:300
SOX9, Goat, R&D systems, AF3075, polyclonal, AB_2194160, 1:300
CPA1, Rabbit, LSBio, LS-C173830, polyclonal, NA ,1:1000
CPA1, Rabbit, Bio-Rad, 1810-0006, polyclonal, AB_616925, 1:1500
NKX2-2, Mouse, DSHB Hybridoma, 74.5A5, 74.5A5, AB_531794, 1:500
NKX2-2, Mouse, abcam, ab187375, NX2/294, AB_2889166, 1:500
Neurogenin 3, Rabbit, Aviva Systems Biology, OACD05949, polyclonal, NA, 1:200
Neurogenin 3, Sheep, R&D Systems, AF3444, polyclonal, AB_2149527, 1:200
NKX6-1, Mouse, DSHB Hybridoma, F55A10, F55A10, AB_532378, 1:300
NKX6-1, Rabbit, Acris/Novus, NBP1-82553, Polyclonal, AB_11023606, 1:500
NKX6-1, Goat, R&D systems, AF5857, Polyclonal, AB_1857045, 1:300
E-Cadherin, Rabbit, Cell Signaling, 3195, 24E10, AB_2291471, 1:200
E-Cadherin, Mouse, TaKaRa, M108, ECCD-2, AB_2895157, 1:400
Beta-catenin, Mouse,BD ,610154, 14, AB_397555, 1:400
Ezrin, Mouse, Abcam, ab4069, 3C12, AB_304261 ,1:300
KRT19, Rat, DSHB, TROMA-III ,TROMA-III, AB_2133570, 1:500
Cytokeratin, Rabbit, Dako (Agilent) Z062201-2, Polyclonal, AB_2650434, 1:500
NFIB, Rabbit, Sigma, HPA003956, Polyclonal, AB_1854424, 1:500
MEIS2, Rabbit, Sigma, HPA003256, Polyclonal, AB_1079356,1:500
NR2F2 Rabbit Abcam ab211776 EPR18442 AB_2893028 1:500
CD326 (EpCAM) PE-Cyanine7 conjugated, NA, LIFE Technologies, 25-5791-80, G8.8, AB_1724047, 1:200

Secondary Antibodies
Target, Species, Fluorophore, Company, Catalogue #, RRID, Dilution
Goat, Donkey, 405, Jackson ImmunoResearch Labs, 705-475-003, AB_2340426, 1:800
Rabbit, Donkey, 405, Abcam, ab175649, AB_2715515, 1:800
Mouse, Donkey, 488, ThermoFisher Scientific, A-21202, AB_141607, 1:800
Goat, Donkey, 488, ThermoFisher Scientific, A-11055, AB_2534102, 1:800
Rabbit, Donkey, 488, ThermoFisher Scientific, A-21206, AB_2535792, 1:800
Rat, Donkey, 488, ThermoFisher Scientific, A-21208, AB_2535794, 1:800
Sheep, Donkey, 488, Jackson ImmunoResearch Labs, 713-546-147, AB_2340746, 1:800

Mouse, Donkey, 555, ThermoFisher Scientific, A-31570, AB_2536180, 1:800
Goat, Donkey, 555, ThermoFisher Scientific, A-21432, AB_2535853, 1:800
Rabbit, Donkey, 555, ThermoFisher Scientific, A-31572, AB_162543, 1:800
Rat, Donkey, 555, Jackson ImmunoResearch Labs, 712-165-153, AB_2340667, 1:800
Sheep, Donkey, 555, ThermoFisher Scientific, A-21436, AB_2535857, 1:800
Mouse, Donkey, 647, Jackson ImmunoResearch Labs, 715-605-151, AB_2340863, 1:800
Goat, Donkey, 647, Jackson ImmunoResearch Labs, 705-605-147, AB_2340437, 1:800
Rabbit, Donkey, 647, ThermoFisher Scientific, A-31573, AB_2536183, 1:800
Rat, Donkey, 647, ThermoFisher Scientific, A-48272, AB_2893138, 1:800
Guinea pig, Donkey, 647, Jackson ImmunoResearch Labs, 706-605-148, AB_2340476, 1:800

Validation

All antibodies were validated for immunocytochemistry and immunohistochemistry analysis by the respective manufacturers.

Eukaryotic cell lines

Policy information about cell lines and Sex and Gender in Research

Cell line source(s)

The parent human embryonic stem cell (hESC) line WA01 (H1) was obtained from WiCell.
The human induced pluripotent stem cell (hiPSC) line HMGUi001-A was generated in house.

Authentication

The pluripotency, trilineage potential, and karyotype of hiPSC line were validated in previous studies (Wang et al. 2018 Mol Metab.; Moya et al. 2020 Stem Cell Res.; Siehler et al. 2020 Stem Cell Res.; Mahaddalkar et al. 2020 Nat Biotechnol.), and hSEC line in previous studies (McCracken et al., 2011 Nat. Protoc.; McGrath et al., 2015 Diabetes; Spence et al., 2011 Nature; Zhang et al. 2019 Dev. Cell).
Pluripotency was confirmed based on typical colony morphology and the expression of pluripotency markers (determined by immunofluorescence, RT-PCR, and/or flow cytometry). Trilineage potential was assessed by the expression of specific markers of the three germ layers following trilineage differentiation (determined by immunofluorescence, RT-PCR, and/or flow cytometry).

Mycoplasma contamination

All cell lines used were tested negative for mycoplasma.

Commonly misidentified lines
(See ICLAC register)

No commonly misidentified lines were used.

Animals and other research organisms

Policy information about studies involving animals; ARRIVE guidelines recommended for reporting animal research, and Sex and Gender in Research

Laboratory animals

Species: Pig;
Breed: German Landrace;
Sample ID, Genotype, Age (embryonic day)
143_susscrofa, Wild-type, 22
MUC11253, Wild-type, 23
MUC11254, Wild-type, 23
139_susscrofa, Wild-type, 33
MUC8384, Wild-type, 40
MUC11250, Wild-type, 54
MUC11251, Wild-type, 54
MUC11252, Wild-type, 54
MUC11260, INS-eGFP, 63
MUC11258, INS-eGFP, 63
MUC11257, Wild-type, 63
MUC11259, Wild-type, 63
MUC12809, Wild-type, 85
MUC12810, Wild-type, 85
MUC12811, INS-eGFP, 85
MUC12812, INS-eGFP, 85
MUC12813, INS-eGFP, 85
MUC12814, INS-eGFP, 85
mT_rest, PTF1A-iCre+/ROSA-mTmG+, 45
mG_only, PTF1A-iCre+/ROSA-mTmG+, 45
mT_mTmG_mG_high, PTF1A-iCre+/ROSA-mTmG+, 85
mG_low, PTF1A-iCre+/ROSA-mTmG+, 85
540_GFP_low, PTF1A-iCre+/ROSA-mTmG+, 63
541_GFP_high, PTF1A-iCre+/ROSA-mTmG+, 63

Wild animals

Not relevant for this study

Reporting on sex	Sex was not considered in this study, as the sex hormones don't have significant effects during embryonic development.
Field-collected samples	Not relevant for this study
Ethics oversight	The use of pigs in this study was approved by the Committee on Animal Health and Care of the local government body of the state of Upper Bavaria in Germany for the wild-type and INS-eGFP German Landrace pigs from the Ludwig Maximilian University of Munich (LMU, Permission No. 55.2-2532.Vet_02-17-136) and the PTF1A-iCre and ROSA-mTmG pigs from the Technical University of Munich (TUM, Permission No. 55.2-2532.Vet_02-18-33). Experiments were performed according to the German Animal Welfare Act and Directive 2010/63/EU on the protection of animals used for scientific purposes.

Note that full information on the approval of the study protocol must also be provided in the manuscript.

Plants

Seed stocks	Not relevant for this study
Novel plant genotypes	Not relevant for this study
Authentication	Not relevant for this study

ChIP-seq

Data deposition

- [x] Confirm that both raw and final processed data have been deposited in a public database such as GEO.
- [x] Confirm that you have deposited or provided access to graph files (e.g. BED files) for the called peaks.

Data access links<br>*May remain private before publication.*	The hESC datasets are accessible through GSE261950 (https://www.ncbi.nlm.nih.gov/geo/query/acc.cgi?acc=GSE261950), GSE261951 (https://www.ncbi.nlm.nih.gov/geo/query/acc.cgi?acc=GSE261951), and GSE261952 (https://www.ncbi.nlm.nih.gov/geo/query/acc.cgi?acc=GSE261952).
Files in database submission	Processed data files<br>run0031_lane9_read1_indexA010-TSLT=9-17-2018input.bw<br>run0031_lane9_read1_indexA001-TSLT=9-17-2018ngn3.bw<br>run0049_lane9_read1_indexA013-NONE=05-04-2019ngn3-30M.bw<br>run0083_lane9_read1_indexU1-U1_07-14-2019ngn3-30M.bw<br>Raw data files<br>run0031_lane9_read1_indexA010-TSLT=9-17-2018input.fastq.gz<br>run0031_lane9_read2_indexA010-TSLT=9-17-2018input.fastq.gz<br>run0031_lane9_read1_indexA001-TSLT=9-17-2018ngn3.fastq.gz<br>run0031_lane9_read2_indexA001-TSLT=9-17-2018ngn3.fastq.gz<br>run0049_lane9_read1_indexA013-NONE=05-04-2019ngn3-30M.fastq<br>run0049_lane9_read2_indexA013-NONE=05-04-2019ngn3-30M.fastq.gz<br>run0083_lane9_read1_indexU1-U1=07-14-2019ngn3-30M.fastq.gz<br>run0083_lane9_read2_indexU1-U1=07-14-2019ngn3-30M.fastq.gz
Genome browser session<br>(e.g. UCSC)	The Genome browser session of the ChIP-seq data itself is not as relevant, since in our study we focused on the gene regulatory network generated by the joint analysis of ChIP, ATAC and RNA-seq data. The raw and processed data is deposit in the GEO.

Methodology

Replicates	3
Sequencing depth	ChIP-seq was performed at a depth of 30M reads per sample
Antibodies	Neurogenin 3, host - sheep, R&D Systems, Cat#AF3444, polyclonal, RRID: AB_2149527
Peak calling parameters	Tag directories were first created from bam files using Homer (v4.11) makeTagDirectory function. Enriched Peaks Regions were called from created tag directories using findPeaks function with factor style, 4 fold enrichment and FDR<0.05. findPeaks <test_TagDirectory> -style factor -o <output_path> -i <input/control_TagDirectory> -fdr 0.05
Data quality	Around 10-13k peaks were identified across the replicates. The peaks were called with Homer factor style, 4 fold enrichment and FDR<0.05 against the input. Peaks from hg19 blacklist region were further removed for downstream analysis.

Software	Bedtools (v2.29.2) and Homer(v4.11) were used for peak calling, motif identification and genomic region overlap. GREAT (v4.0.4) and Homer were used to assign peaks to their nearest coding gene. ChIPseeker(v1.38.0) was used for function annotation, comparison and visualization.
